# The relative clinical impact of sublingual immunotherapy with carbamylated monomeric allergoids on allergic respiratory symptoms

**DOI:** 10.1186/1939-4551-8-S1-A37

**Published:** 2015-04-08

**Authors:** Enrico Compalati, Gianni Mistrello

**Affiliations:** 1 Allergy and Respiratory Diseases Clinic, Italy; 2 Lofarma S.p.a., Italy

## Background

To assess the cumulative and allergen-specific magnitude of effect, and its relative clinical impact, of sublingual allergy immunotherapy with carbamylated monomeric allergoids for allergic rhinoconjunctivitis. Carbamylated allergoids are hypoallergenic extracts with enhanced bioavailability, consequent to a partial resistance to enzymatic degradation, providing efficient dose-tuning.

## Methods

Literature was searched for double-blind placebo-controlled randomized trials administering this treatment, without any restrictions. Pooled analysis of the effects on total symptoms scores (TSSs) during the first season of treatment was performed and for each selected trial the relative clinical impact (RCI) was calculated as the percentage reduction in TSSs obtained with active treatment compared to placebo. The effect size (SMD), the overall and allergen-related RCI were used to estimate an indirect comparison with pharmacotherapy (based on summary data recently extracted from literature).

## Results

Eight studies (4 in grass, 2 in mites, 1 in pellitory, 1 in ragweed allergy) met inclusion criteria and were pooled, comparing overall 180 patients treated with active and 155 with placebo. The overall SMD for TSSs resulted [-0.99 (IC -1.41 to -0.57) p<0.001] with a significant interstudy heterogeneity (I^2^ = 68%). SMD for grass pollen tablets was [-0.58 (IC -0.89 to -0.27) p<0.001], for mite tablets was [-1.54 (IC -2.07 to -1.02) p<0.001]. The weighted mean RCI was overall -31.69%, for grass pollen tablets -32.14%, for mite tablets -24.27%.

## Conclusions

Indirectly compared to the relative clinical impact of standard pharmacotherapy (antihistamines RCI: -15.0%; antileukotrienes RCI: -6.5%; nasal corticosteroids RCI: -23.5%), carbamylated monomeric allergoids result highly effective in providing symptoms relief in seasonal and perennial allergic rhinoconjunctivitis. This estimation is most likely conservative and apparently reduced by the numerous methodological aspects that differentiate clinical trials on drugs and allergen immunotherapy.

